# ENGAGING PEOPLE LIVING WITH DEMENTIA, CARE PARTNERS, AND STAFF TO INFORM REMOTE ME AND MY WISHES

**DOI:** 10.1093/geroni/igae098.0159

**Published:** 2024-12-31

**Authors:** Gail Towsley

**Affiliations:** University of Utah, Salt Lake City, Utah, United States

## Abstract

Me & My Wishes, is a video recording approach for documenting, viewing, listening, and discussing nursing home (NH) residents’ preferences for daily and end-of-life care. This project addressed the alignment and measurement domains of the Readiness Assessment Pragmatic Trial Model. Using a qualitative descriptive design, we conducted focus groups with experts representing persons living with dementia (PLWD) (n=6), care partners (n=8), and NH staff (n=9) to inform us of what mattered to them when implementing and evaluating a remote format of Me & My Wishes. We coded and analyzed audio-recorded transcripts, resolved coding discrepancies in team meetings, and derived domains from finalized codes. Three domains for implementing Me & My Wishes remotely was shared across groups. Prioritizing Care Conferences included leadership providing staff resources and support to prioritize care conferences utilizing Me & My Wishes. Addressing Competing Demands that Prevent Key People Attending involved identifying times for care conferences that allow residents, care partners, and staff to participate. Utilizing Technology and Learning to Communicate in a Virtual Environment encompassed recommendations to have a trained facilitator whom engages attendees and additional support (e.g. technical, emotional) for the PLWD. Experts provided input on content, structure, and design of a template for capturing video highlights, changes in preferences, and results of conversations that could be included in the medical record. Outcomes most important to them included person-centered care, dignity, feeling heard and understood, and improved knowledge. Experts identified key alignment and measurement aspects that informed modifications for delivering Me & My Wishes remotely.

